# Detection of pathogenic bacteria in retailed shrimp from Bangladesh

**DOI:** 10.1002/fsn3.4260

**Published:** 2024-06-17

**Authors:** Murshida Khan, Md. Mahbubur Rahman, Sulav Indra Paul, Julie Anderson Lively

**Affiliations:** ^1^ Department of Fisheries Technology Bangabandhu Sheikh Mujibur Rahman Agricultural University Gazipur Bangladesh; ^2^ Institute of Biotechnology & Genetics Engineering (IBGE) Bangabandhu Sheikh Mujibur Rahman Agricultural University Gazipur Bangladesh; ^3^ School of Renewable Natural Resources Louisiana State University Agricultural Center Baton Rouge Louisiana USA

**Keywords:** farmed shrimp, food safety, pathogenic bacteria

## Abstract

The presence of pathogenic bacteria is a problem that might be present in farmed shrimp due to exposure in the environment or post‐harvest handling. Retail farmed shrimp in Bangladesh (*Penaeus monodon* and *Macrobrachium rosenbergii*) were tested for common pathogenic bacteria namely *Salmonella, L. monocytogenes, Vibrio* spp., and *E. coli*. None of these bacteria were found and instead *Enterobacter cloacae, Escherichia fergusonii, Proteus penneri, Klebsiella aerogenes, Enterococcus faecalis, Serratia marcescens, Citrobacter freundii*, and *Aeromonas dhakensis* were detected. Pathogenic bacteria found in Bangladeshi shrimp may be due to the farm environment, poor handling during harvest or post‐harvest, or unhygienic market conditions. The results indicate that retail shrimp from Bangladesh have food safety concerns. Proper laws and policies need to be enforced and implemented to ensure food safety related to fish and shrimp.

## Graphical abstract

Pathogenic bacteria *Enterobacter cloacae, Escherichia fergusonii, Proteus penneri, Klebsiella aerogenes, Enterococcus faecalis, Serratia marcescens, Citrobacter freundii*, and *Aeromonas dhakensis* were found in Bangladeshi farmed shrimp (*Penaeus monodon* and *Macrobrachium rosenbergii*) collected from retail fish market. The incidence of pathogenic bacteria in retail Bangladeshi shrimp may be due to the farm environment, poor handling during harvest or post‐harvest, or unhygienic market conditions. The results indicate that retail shrimp from Bangladesh have food safety concerns.

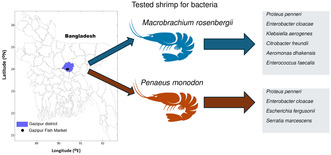

## 1 INTRODUCTION

Microbiological foodborne infection and illness are closely related with food safety, and pathogenic microbial contamination from food is nowadays a great concern. The severity of contamination varies with the amount of the ingestion of contaminated food and individual susceptibility to that microorganisms (Clarence et al., 2009). The microorganisms are naturally present in food, but they have become critical due to their opportunistic pathogenic characteristics (Mhango et al., 2010). Different types of bacterial illness happen due to seafood consumption, and contamination occurs either from the source or from the processing and retail chain (Børresen, 2008). According to the Centers for Disease Control and Prevention (CDC) report, in 2014–2015, around 53% of the seafood outbreaks in the USA were caused by bacteria and 37% by viruses (Centers for Disease Control and Prevention, 2017).

Pathogens transmission occur from fish to humans by contacting an infected person in a related environment, and transmission may depend on the season, handling of fish, food habits of an individual person, and cross‐contamination (Novotny et al., 2004). Shrimp is one of the most consumed seafoods in the world. The presence of pathogenic bacteria can occur in wild and farmed shrimp, and this presence can be a serious concern for food safety if the product is improperly handled, cross‐contamination, or undercooked. Foodborne disease outbreaks from shrimp and fish are well documented (Cuéllar‐Anjel et al., 2010).

The bacterial pathogens present in seafood are *Salmonella* spp., *Aeromonas* spp., *Vibrio* spp. (*V. cholerae*, *V. vulnificus, V. parahaemolyticus*), pathogenic *Escherichia coli*, *Streptococcus iniae, Listeria monocytogenes*, *Clostridium botulinum*, *Clostridium perfringens, Mycobacterium* spp., *Campylobacter jejuni, Edwardsiella tarda*, and *Plesiomonas shigelloides* (Novotny et al., 2004).

Worldwide, *Salmonella* is the leading bacterial reason of fish‐associated disease outbreaks, and in the EU, the second leading cause of foodborne disease is *Salmonella* (European Food Safety Authority, and European Centre for Disease Prevention and Control, 2011; Sheng & Wang, 2021). It is not a natural inhabitant of fish, however; found in various sources such as farm to retail stores, fish skin, fish intestines, raw fish and fishery products, and imported and domestic fishes, and it is introduced to fish by cross‐contamination through contaminated water and improper handling (Fernandes et al., 2018; Sheng & Wang, 2021). *Salmonella* is responsible for salmonellosis disease, and symptoms include abdominal pain, nausea, diarrhea, vomiting, and fever (Jajere, 2019; Ray & Bhunia, 2007).

*Vibrio* spp. are a natural microflora of aquatic environments, and worldwide pathogenic *Vibrio* is a public health concern for the fish consumer, as foodborne infection occurs by consumption of raw and undercooked seafood and the presence of any pathogenic *Vibrio* is the reason for rejection in international trade and import bans (Gopal et al., 2005; Messelhausser et al., 2010; WHO, 2020). Among *Vibrio* spp., the most important human pathogens are *V. cholerae, V. vulnificus*, and *V. parahaemolyticus* as they are responsible for gastrointestinal illnesses or septicemia that might lead to fatal complications (Faruque & Nair, 2006). *V. cholerae* is of most concern as it is the reason cholera makes it globally a public threat and immune‐compromised patients can be killed by a *V. vulnificus* infection (Harwood et al., 2004).

Outbreaks of listeriosis occur due to consumption of animal‐based foods (USFDA, 2018), especially mussels, shrimp, and undercooked seafood (Norhana et al., 2010), and this species is isolated frequently from different fishery products worldwide (Parihar et al., 2008). *L. monocytogenes* has been recognized as a human pathogen since 1929, and it is responsible for foodborne infection with high mortality rates, termed listeriosis (Embarek, 1994; McLauchlin et al., 2004). *L. monocytogenes* can be found in captured fish of contaminated water and also contamination happened during transportation and environment in fish market (Jamali et al., 2015).

*Escherichia coli* indicates fecal contamination and causes health problems like diarrhea, kidney and bladder infections, dysentery, and hemolytic uremic syndrome, depending on the strains (Ray & Bhunia, 2007). *E. coli* is an indicator of fecal contamination, and enterotoxigenic *E. coli* outbreaks have occurred due to consumption of contaminated butterfly shrimp in a sushi restaurant (Jain et al., 2008). *E. coli* O157:H7 was found in shrimp *Fenneropenaeus indicus*, also due to unhygienic handling practices (Surendraraj et al., 2010). Seafood from the Indian Ganges delta found widespread occurrences of *V. parahaemolyticus, V. cholera*, and *E. coli* (Saha et al., 2020).

In Bangladesh, shrimp is the second largest export product, where most are farmed, and two main species are black tiger shrimp (*Penaeus monodon*, Fabricius, 1798) and giant freshwater prawn (*Macrobrachium rosenbergii*, de Man, 1879) (Karim et al., 2019; Khan & Lively, 2023). In 2022, around 251,964 MT of shrimp were produced in Bangladesh, where farmed shrimp was 55.34% (DoF, 2022). For exporting shrimp, the processing plant of Bangladesh always follows the international rules for maintaining shrimp quality such as bacterial status, antibiotic residue, or the presence of filth and dirt. On the other hand, shrimp used to sell in the domestic market of Bangladesh are not checked for bacterial quality as well as antibiotic residues. In the US, European Union (EU), Australia, New Zealand, and Hong Kong, the legal limit for *Salmonella, Listeria monocytogenes*, and *Vibrio cholerae* in 25 g of raw or cooked shrimp is zero (Norhana et al., 2010). In 2018 and 2019, shrimp from Bangladesh, India, and Indonesia that were imported into the US were rejected due to the presence of *Salmonella* spp. (USFDA, 2018, 2019). On the other hand, the status of *Salmonella, L. monocytogenes, Vibrio* spp., *E. coli*, and identification and isolation of bacteria from retail pangas and farmed shrimp have already been reported (Khan et al., 2022, 2023). However, the microbiological status of shrimp found in the retail market in Gazipur, Bangladesh, has not been reported yet for food safety purposes. For the above reasons, this study aimed to investigate the bacterial quality (occurrence of *Salmonella, V. cholerae, E. coli*, and other pathogenic bacteria) of fresh shrimp from fish markets of Gazipur of Bangladesh available for retail purchase for human consumption.

## 2 MATERIALS AND METHODS

### 2.1 Sampling area and sample collection

**FIGURE 1:**
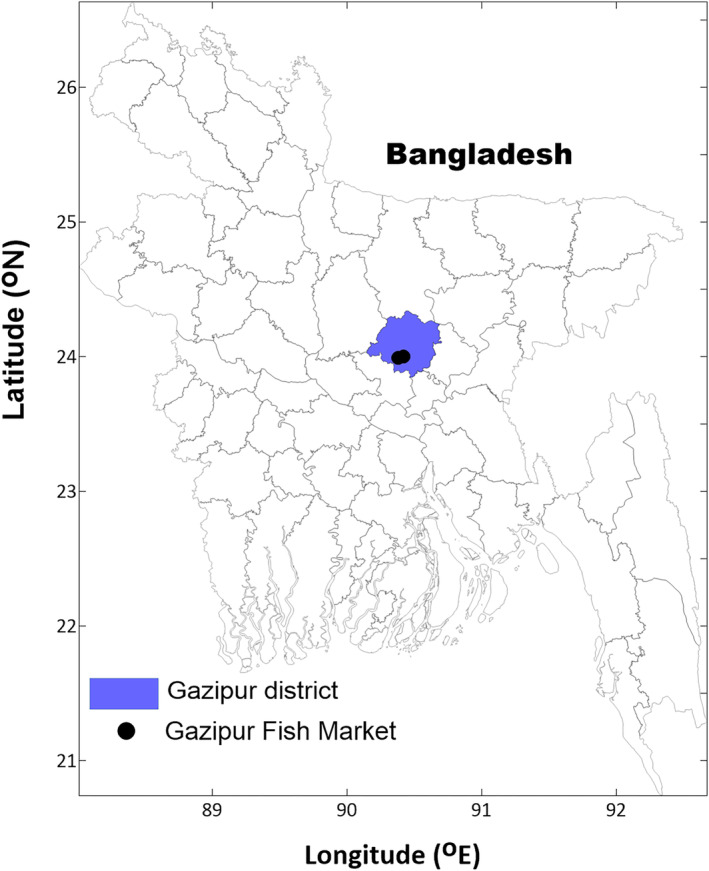
Gazipur district and fish market location.

All the samples were collected from the Gazipur fish market during the fall of 2017. Three times randomly, both freshwater prawn (*Macrobrachium rosenbergii*) and iced Asian tiger shrimp (*Penaeus monodon*) were collected. Three replicates of each of the species were collected, and each time, 20 shrimp samples were collected (*n* = 120 samples, 60 *Macrobrachium rosenbergii* and 60 *Penaeus monodon* shrimp). Iced shrimp samples were transported to the Fisheries Technology lab at the Bangabandhu Sheikh Mujibur Rahman Agricultural University (24.0360° N, 90.3962° E), Gazipur (Figure 1). After reaching the lab, the shrimp were kept at −20°C until analysis. Frozen shrimp were thawed within 12 h at 2–5°C.

### 2.2 Isolation procedure for *Salmonella* spp., *Vibrio* spp., *Escherichia coli*, *Listeria monocytogenes*, and other pathogenic bacteria

*Salmonella, Vibrio*, and *Listeria monocytogenes* testing followed the standard Bacteriological Analytical Manual (Andrews et al., 2000; Hitchins et al., 2017; Kaysner & DePaola Jr, 2004) and *Escherichia coli* determination followed the petrifilm method (3 M™ Petrifilm™ *E. coli*). From each shrimp sample replicate (*n* = 12), five replicates were randomly selected for testing (*n* = 60 per bacteria test). In brief, for *Salmonella*, a 25 g shrimp sample was mixed with sterile 225 mL lactose broth and incubated for 60 ± 5 min at room temperature and then incubated for 24 ± 2 h at 35°C. After incubation, 0.1 mL mixture was added to 10 mL Rappaport‐Vassiliadis (RV) medium and vortexed. RV medium was incubated in a water bath at 42°C for 24 h. After incubation, the RV medium was vortexed, and a 3 mm loopful of RV broth was streaked on xylose lysine desoxycholate or XLD (Sigma) agar. Incubation of the XLD petri dish was done for 24 h at 35°C. After incubation, petri dishes were examined for the presence of *Salmonella* colonies. The susceptive colonies were streaked on nutrient agar to isolate a single colony or pure culture. Incubation of the nutrient agar (NA) petri dish was done for 24 h at 35°C. The colonies from the NA were inoculated in triple sugar iron agar (TSI, Himedia‐M021) slants and streaked and stabbed into lysine iron agar (LIA, Himedia‐M377) slants and incubated at 35°C for 24 h. For *V. cholera* and *V. vulnificus*, a 25 g shrimp sample was mixed with sterile 225 mL alkaline peptone water, blended, and homogenized mixture incubated for 24 h at 35°C. A 3 mm loopful from the surface of the sample solution was streaked on thiosulfate‐citrate‐bile salts‐sucrose (TCBS) agar (Sigma). Incubation of the TCBS petri dish was done for 18–24 h at 35°C. After incubation, the petri dishes were examined for the presence of *V. cholerae* and *V. vulnificus* colonies. For *E. coli*, a 25 g shrimp sample was mixed with sterile phosphate‐buffered saline (PBS) (225 mL), blended, and solutions were prepared up to 10^−4^ dilutions. Around 1 mL diluted sample was placed in the middle of the 3 M petrifilm *E. coli* coliform count plate. After a minimum of 1 min, wait for the gel to solidify and the petrifilm was incubated at 36°C for 18–24 h. For *L. monocytogenes*, a 25 g sample was mixed with sterile UVM listeria enrichment broth (225 mL), blended, and incubated at 30°C for 2 days. After incubation, 1 mL of enrichment broth was plated on oxford medium and incubated at 37°C for 2 days. The red colonies from the petrifilm (thought to be total coliform), the yellow colonies (oxidase positive) from XLD agar, and the oxidase negative yellow and green colonies from TCBS agar were chosen in order to identify other bacteria besides *E. coli*, *Salmonella* spp., and *Vibrio* spp. Prior to molecular identification, such isolates were routinely sub‐cultured on NA.

### 2.3 DNA extraction and quantitative PCR

Pure cultures were kept in nutrient broth with 10% glycerol and stored at −20°C for molecular identification. The susceptive bacterial colonies were taken from pure culture stock, inoculated into a nutrient broth (Liofilchem), and incubated in a shaker incubator (120 rpm) at 28°C for 24–48 h. GeneJET Genomic DNA purification kit (Thermo Scientific, # K0721) was used for DNA extraction following the manufacturer's protocol. Electrophoresis was used to check the DNA quality by comparing it with the 1Kb plus DNA ladder marker (Thermo Fisher Scientific, USA). The quantity of the extracted DNA was estimated after electrophoresis in 0.8% agarose gel compared with the concentrations of lambda DNA marker (Promega) (Hannan et al., 2019). The polymerase chain reaction (PCR) with 8F and 1492R universal primer sets (Macrogen, Korea) was used for amplification (Paul, 2018; Khan & Lively, 2023). The PCR mixture reagents were mixed according to Hannan et al. (2019) with modification in primer concentrations (Table 1). The PCR thermocycler (2720 thermal cycler, Applied Biosystems) was used for amplification. The thermal profile of PCR was an initial dilution step set up for 5 min at 94°C, a denaturation step of 35 cycles at 94°C for 1 min, annealing for 40 s at 57°C, extension step for 1 min at 72°C, and final extension step for 10 min at 72°C. The PCR product was purified using a commercial PCR Purification Kit (Thermo Scientific GeneJET PCR Purification Kit #K0701) and after purification, the purified PCR product was stored at −20°C for further use. The purified PCR products with sequencing primer were sent to the National Institute of Biotechnology, Savar, Dhaka, for sequencing of the 16S rRNA gene. The sequence results were compared to BLAST (Basic Local Alignment Search Tool) at the National Center for Biotechnology Information website (NCBI, http://www.ncbi.nlm.nih.gov/) (Rahman et al., 2017).

**TABLE 1:** The concentration of polymerase chain reaction mixture.

Reagents	Concentration	Volume (μL)
25 mM MgCl_2_ (Thermo Fisher Scientific)	1.5 mM	6
10 × PCR buffer (Thermo Fisher Scientific)	1 X	10
10 mM dNTP (Thermo Fisher Scientific)	200 μM each dNTP	2
F primer (Macrogen Korea)	0.1–1.0 μM	3
R primer (Macrogen Korea)	0.1–1.0 μM	3
DNA template	40‐50 ng/100 μL	5
*Taq* polymerase (Thermo Fisher Scientific)	0.05 U	1
Sterile deionized water		70

## 3 RESULTS

*Salmonella, L. monocytogenes*, *E. coli*, and *Vibrio* spp. were not found in shrimp collected at the local markets in Bangladesh. However, *Proteus penneri, Klebsiella aerogenes, Enterococcus faecalis, Escherichia fergusonii*, *Serratia marcescens, Enterobacter cloacae, Citrobacter freundii*, and *Aeromonas dhakensis* were isolated. *P. penneri* was isolated from both *P. monodon* (100% of samples; 9 isolates) and *M. rosenbergii* (67% of samples; 10 isolates). *E. cloacae* was also found in both shrimp species: 100% of *M. rosenbergii* samples (6 isolates) and 67% of *P. monodon* samples (5 isolates). *K. aerogenes* was isolated from 33% of *M. rosenbergii* samples (1 isolate), the same as *C. freundii*, which was isolated from 33% of *M. rosenbergii* samples (4 isolates). *P. monodon* was also contaminated with *E. fergusonii*, in 33% of samples (2 isolates). *A. dhakensis* was isolated from *M. rosenbergii*, in 33% of samples with five isolates. *E. faecalis* was isolated from 33% of *M. rosenbergii* samples (4 isolates). *S. marcescens* was isolated from 33% of *P. monodon* samples (1 isolate) (Table 2).

**TABLE 2:** Bacteria isolated from the *M. rosenbergii* and *P. monodon*.

Shrimp source	Bacterial species	Isolates	Replicates found (out of 3)	Accession number
*M. rosenbergii*	*Proteus penneri*	10	2	MN262114
	*Enterobacter cloacae*	6	3	MN262110
	*Klebsiella aerogenes*	1	1	MN262189
	*Citrobacter freundii*	4	1	MN262188
	*Aeromonas dhakensis*	5	1	MN262119
	*Enterococcus faecalis*	4	1	MN262191
*P. monodon*	*Proteus penneri*	9	3	–
	*Enterobacter cloacae*	5	2	MN262185
	*Escherichia fergusonii*	2	1	MN262113
	*Serratia marcescens*	1	1	MN262186

## 4 DISCUSSION

While no *E. coli*, *Salmonella, V. cholerae, V. vulnificus*, and *L. monocytogenes* were detected in shrimp, eight different species were detected: *P. penneri, K. aerogenes, E. faecalis, E. fergusonii*, *S. marcescens, E. cloacae, C. freundii*, and *A. dhakensis*. These findings have food safety implications.

*P. penneri* is included in the Enterobacteriaceae family, and it is an invasive pathogen (Kishore, 2012). *P. penneri* was found in *Macrobrachium rosenbergii* shrimp and sediments of both *Macrobrachium rosenbergii* and *Penaeus monodon* shrimp farm ponds in Bangladesh (Khan et al., 2022). Therefore, shrimp farms could be a source of *P. penneri* entering the seafood chain. This species is a destructive agent of farmed shrimp as it is responsible for causing red body disease of white shrimp *Penaeus vannamei*, and significant economic losses occurred in several Southeast and East Asian countries due to this disease outbreak (Cao et al., 2014; Matyar, 2017; Zheng et al., 2015). In addition to causing disease in shrimp, it is also a known pathogen in fish (Mandal et al., 2002) and humans (Kishore, 2012). The presence of this species in shrimp is a bad indicator as it has been isolated from urine, blood cultures and generally infects abdominal wounds, urinary tract, groin, ankle, and blood (Kishore, 2012).

*E. cloacae* is a gram‐negative bacterium of the Enterobacteriaceae family, a common bacterium that lives in the gut microbiota in healthy humans and is considered opportunistic pathogen responsible for nosocomial infections (Davin‐Regli, 2015; Leong et al., 2017). In Vietnam, *E. cloacae* complex was isolated from farm‐originated *Penaeus monodon* (Asian tiger shrimp) and *P. vannamei* (White leg shrimp) (Brouwer et al., 2018). This species was also found in Brazil, isolated from farmed shrimp *Litopenaeus vannamei* and farmed fish *Oreochromis niloticus* (Almeida et al., 2017). In China, *E. cloacae* infection frequently happens in *M. rosenbergii* hatcheries, which is responsible for slow‐growing prawns (Gao et al., 2021). The presence of *E. cloacae* in shrimp indicates that they were not properly handled, and fecal contamination occurred at some point as this species is not a natural microflora in fish and shrimp (Almeida et al., 2017). The possible sources of *E. cloacae* include the water of the shrimp ponds, which may be contaminated by nearby fecal sources, improper handling during harvesting, storage, or during sale in a fish market (Mandal et al., 2009; Roy et al., 2013). The water in Bangladesh can be contaminated by fecal coliforms during the rainy season from various sources, including poor waste management systems, poor sanitary conditions in rural areas, and nearby farms. Fish markets can also be a source of contamination as fish markets in Bangladesh; however, in this study, the origin of *E. cloacae* may be from shrimp farms as in Southeast Asia, and this species was frequently isolated from farmed shrimp and fish (Almeida et al., 2017; Brouwer et al., 2018; Gao et al., 2021).

*K. aerogenes* (species *K. pnemoniae*; subspecies *aerogenes*) is commonly found in water and human feces (Chart, 2012). *K. aerogenes* has also been found in frozen shrimp from Bangladesh (Noor et al., 2015), and in Brazil, it was isolated from farmed shrimp *Litopenaeus vannamei* and farmed fish *Oreochromis niloticus* (Almeida et al., 2017). The presence of *K. aerogenes* in shrimp samples can be considered an alert, and the health risks caused by this strain should not be underestimated. Some studies support the present study since they reported the presence of this species in fish, seafood, and other types of food (Guo et al., 2016; Marathe et al., 2016). *C. freundii* is a commensal and native organism in the intestinal tracts of both animals and humans and causes diarrhea and infection (Liu et al., 2017). This species was isolated from farmed shrimp and farmed tilapia in Brazil, retail shrimp in Iraq, and farmed goldfish in India, which corroborates this study (Almeida et al., 2017; Preena et al., 2021; Qasim, 2023). The presence of *K. aerogenes* in *M. rosenbergii* may be due to poor water quality, the farm environment, or unhygienic handling, and the presence of *C. freundii* is not unusual due to the presence of this organism in freshwater. The eating of *K. aerogenes* and *C. freundii*‐contaminated shrimp can create adverse effects on human health.

*E. fergusonii* is a gram‐negative bacterium of the Enterobacteriaceae family, which is considered an emerging human pathogen and has been previously isolated in Benin from the shrimp *P. notialis* (Dabadé, 2015). In Iraq, *E. fergusonii* was isolated from *Cyprinus carpio* (Mohammed et al., 2023). *E. fergusonii*, a member of the genus *Escherichia*, has been reported to transmit via the food chain and cause diseases in humans. *E. fergusonii* has been isolated from several clinical cases, such as bacteremia, wound infections, urinary tract infections, and diarrhea, which indicate that it can be responsible for diseases and infections (Adesina et al., 2019; Lai et al., 2011). Several studies suggest that *E. fergusonii* is potentially widespread in the food chain as it was isolated from healthy chickens, some Southeast Asian farmed animals and associated with pneumonia in a beef cow (Oh et al., 2012; Rimoldi & Moeller Jr., 2013; Rayamajhi et al., 2011). So it is evident that *E. fergusonii* can be contaminated from various sources, and it could pose a potential risk to food safety and public health.

Black spot necrosis of *M. rosenbergii* is caused by *Aeromonas* spp. (Brady & De La Vega, 1992). In Tamil Nadu, India, *Aeromonas* spp. was isolated from *P. semisulcatus* shrimp stored on ice at a fish market (Lakshmanan et al., 2002). The genus *Aeromonas* is also considered both human and fish pathogens that are found in aquatic environments, soil, and animal feces (Rahimi et al., 2014; Rahman et al., 2005; Ray & Bhunia, 2007). The presence of *Aeromonas* spp. in shrimp can be a risk to human health; *A. dhakensis* is responsible for wound infections, bacteremia, and gastroenteritis like other *Aeromonas* spp. (Chen et al., 2016). It has been reported that *A. dhakensis, A. hydrophila*, and *A. jandaei* were isolated from freshwater crocodiles (*Crocodylus siamensis*), suffered from pneumonia and septicemia (Pu et al., 2019). *A. dhakensis* was first isolated from the feces of children suffering from diarrhea in Bangladesh (Huys et al., 2002), and this species has usually been described from human feces, clinical sources, diseased fish, and from water in Asia, Africa, Europe, Mexico, and Australia (Aravena‐Roman et al., 2014; Esteve et al., 2012, 2015; Ghenghesh et al., 2014; Huys et al., 2002; Puah et al., 2013; Soto‐Rodriguez et al., 2013; Wu et al., 2015). Recently, *A. dhakensis* was associated with fatal hemorrhagic necrotizing pneumonia and sepsis in a Risso's dolphin (*Grampus griseus*) from the Mediterranean Sea in Spain, increasing the number of potential hosts of this species (Pérez et al. 2015).

*Enterococcus* spp. is common in seawater and shrimp. In Benin, *E. faecalis* was isolated from shrimp *P. notialis* (Dabadé, 2015). In comparison with the present research, *E. faecalis* has been isolated from shrimp and shrimp ponds in Thailand, Vietnam, Bangladesh, and Poland (Chajęcka‐Wierzchowska et al., 2016; Lohalaksanadech & Sajarit, 2015). In Switzerland, *E. faecalis* has been isolated from shrimp, oysters, pangas, and salmon fish (Boss et al., 2016), and in Iran, isolated from fish, shrimp, and lobster, where some isolates showed resistance toward antibiotics (Noroozi et al., 2022). Sediments and seawater were the primary sources of *E. faecalis* strain contamination in seafood (Di Cesare et al., 2013; Do Vale Pereira et al., 2017; Novais et al., 2018). However, human‐induced cross‐contamination during fishing, storage, transportation, and sale is presented as a risk factor for the presence of *E. faecalis* in seafood samples (Shikongo‐Nambabi, 2011). The higher prevalence of *E. faecalis* in shrimp may be due to lower hygienic conditions and cross‐contamination. Therefore, caution is suggested concerning the origin of isolated *E. faecalis*. The studied samples were bought at retail, and as *E. faecalis* is not a natural bacterial flora of shrimp, its high prevalence may indicate that human and animal fecal contamination of the aquaculture environment occurred during processing.

*S. marcescens* is an opportunistic pathogen commonly found in soil and water and an important fish pathogen isolated from the aquaculture environment (Adeyemi et al., 2022; Buckle, 2015). It can be transmitted by direct contact, and it is highly pathogenic to humans; it is associated with endocarditis, osteomyelitis, urinary and respiratory infections, septicemia, eye infections, wound infections, and meningitis (Gurevitch & Weber, 1950; Buckle, 2015). *S. marcescens* was previously isolated from seafood in Spain (Böhme et al., 2011) from *Oreochromis niloticus* in Brazil (Almeida et al., 2017). The presence of *S. marcescens* in shrimp is not unusual as it was isolated from aquaculture previously; however, due to its pathogenic nature, it is a threat to humans.

The bacteria contamination found in shrimp could come from the farm environment. In Bangladesh, around 70% of the fresh *M. rosenbergii* was contaminated with fecal coliform (Rahman et al., 2012). The findings of this research are important when considering the safety of consumers in Bangladesh as well as consumers in importing countries. During entry into any importing country, shrimp are tested for bacterial contamination. *Salmonella* has been found, and recently, shrimp shipments were rejected due to the presence of *Salmonella* (USFDA, 2019). The care and treatment of shrimp destined for export are not necessarily the same as that of shrimp destined for domestic markets. In Bangladesh, exported shrimp are handled and processed differently with more care to avoid any objection or rejection from the importing country. For shrimp destined for export, extra care is taken from farm selection to transportation to processing by the processing plant owner. Around 2200 individual quick frozen (IQF) shrimp (including peeled, head‐on, raw, and cooked) destined for export were tested, and only 30 shrimp contained *E. coli*, and it was absent in cooked shrimp (Hatha et al., 2003). Proper washing, proper hygiene in the processing plant, and low‐temperature storage can also reduce the bacterial load.

The scenario is different for local shrimp. From selling to transportation around Bangladesh, there is a high chance of cross‐contamination by unwanted bacteria. Fish markets often do not have hygienic conditions. Therefore, local people in Bangladesh are more at risk for bacterial contamination. At the farm level, good aquaculture practices should be applied to all shrimp, regardless of the final destination, including good water quality, regular water exchange, and proper handling during harvesting, transportation, and storage. After purchase, people should properly wash and cook all seafood. Good aquaculture practices, proper handling, and awareness education among farmers and consumers can decrease the chance of contamination by harmful bacteria.

Additionally, innovations in shrimp packaging and antimicrobial treatments or compounds could be beneficial (Tsironi & Taoukis, 2018). While modified atmosphere packaging and other advances might be practical in the US or for shrimp destined for export, many of these packaging techniques are cost‐prohibitive and not feasible, especially for local markets. The use of natural extracts could be practical and beneficial for local markets in countries like Bangladesh. Compounds like pomegranate peel extracts or key lime juice have been found to limit microbial growth in shrimp (Ismail et al., 2019; Velu et al., 2019). Future research on accessible, local compounds that might reduce the bacterial load in seafood could have a significant impact on food safety.

## 5 CONCLUSIONS

Local farm‐raised shrimp (*P. monodon* and *M. rosenbergii*) from Bangladesh collected from the retail fish market of Gazipur were tested for harmful human bacteria, including *Salmonella, E. coli, V. cholerae, V. vulnificus*, *L. monocytogenes*, and other pathogenic bacteria. None of the five specific bacteria were found, but *P. penneri, K. aerogenes, E. faecalis, E. fergusonii*, *S. marcescens, E. cloacae, C. freundii*, and *A. dhakensis* were isolated. So, this research investigated the bacterial quality of retail shrimp in the Gazipur fish market, and this knowledge will be helpful for consumers, distributors, processors, and researchers worldwide. Here, some bacteria are natural microflora in shrimp farms, but the presence of fecal coliform indicated that fecal contamination occurred at some point in the supply chain. The presence of these bacteria indicates that consumers in Bangladesh are at risk for food safety if proper handling is not followed. However, it is critical that all shrimp be handled correctly from harvest to consumption, and that proper hygiene be practiced at all levels. Regulations about good aquaculture practice (GAP) are already present in Bangladesh, but they are difficult to implement and inspect. It should be started at the farm in the form of clean surroundings, water from good sources, good sanitary conditions, and consciousness development of the farmer through proper training. Rough handling is common during transportation from farm to market across Bangladesh. Because many people are involved, like farmers, intermediate handlers or Faria, depot owners, and retailers, maintaining hygiene is critical. Handling from the farm to market needs to be reduced, good quality ice supply should be ensured, clean ice boxes are needed instead of baskets or pots, good infrastructure in the selling area or fish market, and finally increasing awareness of cleanliness among farmers, transporters, and sellers is needed. Proper laws and policies need to be enforced and implemented to ensure food safety related to fish and shrimp. Considering the presence of pathogenic bacteria in retail shrimp, in the future the source needs to be identified. Bangladeshi People may suffer from different diseases due to consuming contaminated shrimp without knowing the reason for the disease. Although this study showed only the scenario of the sampling area, it indicates that fish from other areas may also be contaminated by different pathogenic bacteria, and it is a major threat to food safety concern. Therefore, other areas of shrimp and fish need to be examined for the presence of pathogenic bacteria.

## AUTHOR CONTRIBUTIONS

**Murshida Khan:** Conceptualization (equal); data curation (equal); formal analysis (equal); investigation (equal); methodology (equal); validation (equal); visualization (equal); writing – original draft (equal); writing – review and editing (equal). **Md. Mahbubur Rahman:** Conceptualization (supporting); formal analysis (equal); investigation (equal); methodology (equal); validation (equal); visualization (equal); writing – review and editing (equal). **Sulav Indra Paul:** Formal analysis (equal); investigation (equal); methodology (equal); visualization (equal); writing – review and editing (equal). **Julie Anderson Lively:** Conceptualization (equal); formal analysis (equal); funding acquisition (equal); investigation (equal); methodology (equal); project administration (equal); resources (equal); supervision (equal); validation (equal); visualization (equal); writing – review and editing (equal).

## CONFLICT OF INTEREST STATEMENT

The authors declare no conflict of interest.

## Data Availability

The datasets 16S Ribosomal RNA genes (Accession number in Table 2) for this study can be found in the GenBank https://www.ncbi.nlm.nih.gov/nuccore/. Accessed on 6 August 2019.

## References

[fsn34260-bib-0001] Adesina, T. , Nwinyi, O. , De, N. , Akinnola, O. , & Omonigbehin, E. (2019). First detection of carbapenem‐resistant *Escherichia fergusonii* strains harbouring beta‐lactamase genes from clinical samples. Pathogens, 8, 164. 10.3390/pathogens8040164 31557915 PMC6963453

[fsn34260-bib-0002] Adeyemi, J. A. , Nwanze, J. , & Adedire, C. O. (2022). Evaluation of hemato‐immune parameters in African catfish, *Clarias gariepinus* (Burchell 1822) experimentally challenged with *Serratia marcescens* . Comparative Clinical Pathology, 31(3), 475–481.

[fsn34260-bib-0003] Almeida, M. V. A. D. , Cangussú, Í. M. , Carvalho, A. L. S. D. , Brito, I. L. P. , & Costa, R. A. (2017). Drug resistance, AmpC‐β‐lactamase and extended‐spectrum β‐lactamase‐producing Enterobacteriaceae isolated from fish and shrimp. Revista Do Instituto de Medicina Tropical de São Paulo, 59, e70. 29116290 10.1590/S1678-9946201759070 PMC5679682

[fsn34260-bib-0004] Andrews, W. H. , Wang, H. , Jacobson, A. , & Hammack, T. (2000). Laboratory methods ‐ bacteriological analytical manual (BAM) chapter 5: Salmonella. https://www.fda.gov/Food/FoodScienceResearch/LaboratoryMethods/ucm070149.htm

[fsn34260-bib-0082] Aravena‐Román, M. , Inglis, T. J. J. , Riley, T. V. , & Chang, B. J. (2014). Distribution of 13 virulence genes among clinical and environmental *Aeromonas* spp. in Western Australia. European Journal of Clinical Microbiology & Infectious Diseases, 33(11), 1889–1895. 10.1007/s10096-014-2157-0 24859908

[fsn34260-bib-0007] Boss, R. , Overesch, G. , & Baumgartner, A. (2016). Antimicrobial resistance of *Escherichia coli*, *Enterococci, Pseudomonas aeruginosa*, and *Staphylococcus aureus* from raw fish and seafood imported into Switzerland. Journal of Food Protection, 79(7), 1240–1246. 27357045 10.4315/0362-028X.JFP-15-463

[fsn34260-bib-0008] Brady, Y. J. , & De La Vega, E. L. (1992). Communications: Bacteria in the Hemolymph of the freshwater prawn *Macrobrachium rosenbergii* . Journal of Aquatic Animal Health, 4(1), 67–69. 10.1577/1548-8667(1992)004<0067:CBITHO>2.3.CO;2

[fsn34260-bib-0009] Brouwer, M. S. , Rapallini, M. , Geurts, Y. , Harders, F. , Bossers, A. , Mevius, D. J. , Wit, B. , & Veldman, K. T. (2018). *Enterobacter cloacae* complex isolated from shrimps from Vietnam carrying bla IMI‐1 resistant to carbapenems but not cephalosporins. Antimicrobial Agents and Chemotherapy, 62(7), e00398‐18. 29686153 10.1128/AAC.00398-18 PMC6021663

[fsn34260-bib-0010] Buckle, J. (2015). Infection. In J. Buckle (Ed.), Clinical aromatherapy: Essential oils in healthcare (Third ed., pp. 130–167). Churchill Livingstone.

[fsn34260-bib-0005] Böhme, K. , Fernández‐No, I. C. , Gallardo, J. M. , Cañas, B. , & Calo‐Mata, P. (2011). Safety assessment of fresh and processed seafood products by MALDI‐TOF mass fingerprinting. Food and Bioprocess Technology, 4(6), 907–918. 10.1007/s11947-010-0441-2

[fsn34260-bib-0006] Børresen, T. (Ed.). (2008). Improving seafood products for the consumer. Elsevier.

[fsn34260-bib-0011] Cao, H. , He, S. , Lu, L. , Yang, X. , & Chen, B. (2014). Identification of a *Proteus penneri* isolate as the causal agent of red body disease of the cultured white shrimp *Penaeus vannamei* and its control with *Bdellovibrio bacteriovorus* . Antonie Van Leeuwenhoek, 105(2), 423–430. 10.1007/s10482-013-0079-y 24271474

[fsn34260-bib-0012] Centers for Disease Control and Prevention (CDC) . (2017). https://www.cdc.gov/foodsafety/pdfs/2015foodborneoutbreaks_508.pdf

[fsn34260-bib-0013] Chajęcka‐Wierzchowska, W. , Zadernowska, A. , & Łaniewska‐Trokenheim, Ł. (2016). Virulence factors, antimicrobial resistance and biofilm formation in *Enterococcus* spp. isolated from retail shrimps. LWT‐ Food Science and Technology, 69, 117–122. 10.1016/j.lwt.2016.01.034

[fsn34260-bib-0014] Chart, H. (2012). *Klebsiella*, enterobacter, proteus and other enterobacteria: Pneumonia; urinary tract infection; opportunist in‐fection. In Medical Microbiology (pp. 290–297). Elsevier Inc.

[fsn34260-bib-0015] Chen, P. L. , Lamy, B. , & Ko, W. C. (2016). *Aeromonas dhakensis*, an increasingly recognized human pathogen. Frontiers in Microbiology, 7, 793. 10.3389/fmicb.2016.00793 27303382 PMC4882333

[fsn34260-bib-0016] Clarence, S. Y. , Obinna, C. N. , & Shalom, N. C. (2009). Assessment of bacteriological quality of ready to eat food (meat pie) in Benin City metropolis, Nigeria. African Journal of Microbiology Research, 3(6), 390–395.

[fsn34260-bib-0017] Cuéllar‐Anjel, J. , Corteel, M. , Galli, L. , Alday‐Sanz, V. , & Hasson, K. W. (2010). Principal shrimp infectious diseases, diagnosis and man‐agement. The Shrimp Book, 517–621.

[fsn34260-bib-0018] Dabadé, D. S. (2015). Shrimp quality and safety management along the supply chain in Benin (Doctoral dissertation. Wageningen University.

[fsn34260-bib-0019] Davin‐Regli, A. (2015). *Enterobacter aerogenes* and *Enterobacter cloacae*; versatile bacterial pathogens confronting antibiotic treat‐ment. Frontiers in Microbiology, 6, 392. 10.3389/fmicb.2015.00392 26042091 PMC4435039

[fsn34260-bib-0020] Di Cesare, A. , Luna, G. M. , Vignaroli, C. , Pasquaroli, S. , Tota, S. , Paroncini, P. , & Biavasco, F. (2013). Aquaculture can promote the presence and spread of antibiotic‐resistant Enterococci in marine sediments. PLoS One, 8(4), e62838. 10.1371/journal.pone.0062838 23638152 PMC3637307

[fsn34260-bib-0091] do Vale Pereira, G. , da Cunha, D. G. , Pedreira Mourino, J. L. , Rodiles, A. , Jaramillo‐Torres, A. , & Merrifield, D. L. (2017). Characterization of microbiota in *Arapaima gigasintestine* and isolation of potential probiotic bacteria. Journal of Applied Microbiology, 123(5), 1298–1311. 10.1111/jam.13572 28833934

[fsn34260-bib-0095] DoF (Department of Fisheries) . (2022). *Motso soptaho* (Bengali version). Department of Fisheries, Ministry of Fisheries and Livestock, Dhaka, Bangladesh. 144.

[fsn34260-bib-0080] Embarek, P. K. B. (1994). Presence, detection and growth of *Listeria monocytogenes* in seafoods: A review. International Journal of Food Microbiology, 23(1), 17–34. 10.1016/0168-1605(94)90219-4 7811570

[fsn34260-bib-0094] Esteve, C. , Alcaide, E. , & Blasco, M. D. (2012). *Aeromonas hydrophila* subsp. *dhakensis* isolated from feces, water and fish in Mediterranean Spain. Microbes and Environments, 27(4), 367–373. 22472298 10.1264/jsme2.ME12009 PMC4103543

[fsn34260-bib-0083] Esteve, C. , Alcaide, E. , & Giménez, M. J. (2015). Multidrug‐resistant (MDR) *Aeromonas* recovered from the metropolitan area of Valencia (Spain): Diseases spectrum and prevalence in the environment. European Journal of Clinical Microbiology & Infectious Diseases, 34, 137–145. 25082185 10.1007/s10096-014-2210-z

[fsn34260-bib-0021] European Food Safety Authority, European Centre for Disease Prevention and Control . (2011). The European Union summary report on trends and sources of zoonoses, zoonotic agents and food‐borne outbreaks in 2009. EFSA Journal, 9(3), 1–378. 10.2903/j.efsa.2011.2090 PMC11926762 40125543

[fsn34260-bib-0022] Faruque, S. M. , & Nair, G. B. (2006). Epidemiology. In F. L. A. B. Thompson & J. Swings (Eds.), The biology of vibrios (pp. 385–398). ASM Press.

[fsn34260-bib-0078] Fernandes, S. A. , Tavechio, A. T. , Ghilardi, Â. C. R. , Almeida, E. A. D. , Silva, J. M. L. D. , Camargo, C. H. , & Tiba‐Casas, M. R. (2022). *Supplementary material of the article "Salmonella enterica serotypes from human and nonhuman sources in Sao Paulo State, Brazil, 2004–2020"* [Data set]. SciELO Data. 10.48331/SCIELODATA.MLW3RU PMC9528755 36197427

[fsn34260-bib-0023] Gao, X. , Zhou, Y. , Zhu, X. , Tang, H. , Li, X. , Jiang, Q. , Wei, W. , & Zhang, X. (2021). *Enterobacter cloacae*: A probable etiological agent associated with slow growth in the giant freshwater prawn *Macrobrachium rosenbergii* . Aquaculture, 530, 735826.

[fsn34260-bib-0084] Ghenghesh, K. S. , Ahmed, S. F. , Cappuccinelli, P. , & Klena, J. D. (2014). Genospecies and virulence factors of *Aeromonas* species in different sources in a North African country. Libyan Journal of Medicine, 9(1), 25497. 10.3402/ljm.v9.25497 25216211 PMC4161726

[fsn34260-bib-0024] Gopal, S. , Otta, S. K. , Kumar, S. , Karunasagar, I. , Nishibuchi, M. , & Karunasagar, I. (2005). The occurrence of vibrio species in tropical shrimp culture environments; implications for food safety. International Journal of Food Microbiology, 102(2), 151–159. 10.1016/j.ijfoodmicro.2004.12.011 15992615

[fsn34260-bib-0025] Guo, Y. , Zhou, H. , Qin, L. , Pang, Z. , Qin, T. , Ren, H. , Pan, Z. , & Zhou, J. (2016). Frequency, antimicrobial resistance and genetic diversity of *Klebsiella pneumoniae* in food samples. PLoS One, 11(4), e0153561. 27078494 10.1371/journal.pone.0153561 PMC4831839

[fsn34260-bib-0026] Gurevitch, J. , & Weber, D. (1950). A strain of Serratia isolated from urine. American Journal of Clinical Pathology, 20(1), 48–49. 15400105 10.1093/ajcp/20.1.48

[fsn34260-bib-0027] Hannan, M. A. , Rahman, M. M. , Mondal, M. N. , Deb, S. C. , Chowdhury, G. , & Islam, M. T. (2019). Molecular identification of *Vibrio alginolyticus* causing Vibriosis in shrimp and its herbal remedy. Polish Journal of Microbiology, 68(4), 429–438. 10.33073/pjm-2019-042 31880887 PMC7260635

[fsn34260-bib-0028] Harwood, V. J. , Gandhi, J. P. , & Wright, A. C. (2004). Methods for isolation and confirmation of *Vibrio vulnificus* from oysters and environmental sources: A review. Journal of Microbiological Methods, 59(3), 301–316. 10.1016/j.mimet.2004.08.001 15488274

[fsn34260-bib-0029] Hatha, A. M. , Maqbool, T. , & Kumar, S. S. (2003). Microbial quality of shrimp products of export trade produced from aquacultured shrimp. International Journal of Food Microbiology, 82(3), 213–221. 10.1016/s0168-1605(02)00306-9 12593924

[fsn34260-bib-0030] Hitchins, A. D. , Jinneman, K. , & Chen, Y. (2017). Laboratory methods – BAM: Detection and Enumeration of Listeria monocytogenes. https://www.fda.gov/food/foodscienceresearch/laboratorymethods/ucm071400.htm

[fsn34260-bib-0031] Huys, G. , Kampfer, P. , Albert, M. J. , Kuhn, I. , Denys, R. , & Swings, J. (2002). *Aeromonas hydrophila* subsp. *dhakensis* subsp. nov., isolated from children with diarrhoea in Bangladesh, and extended description of *Aeromonas hydrophila* subsp. *hydrophila* (Chester 1901) Stanier 1943 (approved lists 1980). International Journal of Systematic and Evolutionary Microbiology, 52, 705–712. 12054229 10.1099/00207713-52-3-705

[fsn34260-bib-0032] Ismail, T. , Suleman, R. , Akram, K. , Hameed, A. , Llah, I. U. , Amir, M. , & Akhtar, S. (2019). Pomegranate (*Punica granatum* L.) Peel extracts inhibit microbial growth and lipid oxidation in minced shrimps stored at 4°C. Journal of Aquatic Food Product Technology, 28(1), 84–92. 10.1080/10498850.2018.1561571

[fsn34260-bib-0033] Jain, S. , Chen, L. , Dechet, A. , Hertz, A. T. , Brus, D. L. , Hanley, K. , Wilson, B. , Frank, J. , Greene, K. D. , Parsons, M. , Bopp, C. A. , Todd, R. , Hoekstra, M. , Mintz, E. D. , & Ram, P. K. (2008). An outbreak of enterotoxigenic *Escherichia coli* associated with sushi restaurants in Nevada, 2004. Clinical Infectious Diseases, 47, 1–7. 10.1086/588666 18491967

[fsn34260-bib-0079] Jajere, S. M. (2019). A review of *Salmonella enterica* with particular focus on the pathogenicity and virulence factors, host specificity and antimicrobial resistance including multidrug resistance. Veterinary World, 12(4), 504–521. 10.14202/vetworld.2019.504-521 31190705 PMC6515828

[fsn34260-bib-0034] Jamali, H. , Paydar, M. , Ismail, S. , Looi, C. Y. , Wong, W. F. , Radmehr, B. , & Abedini, A. (2015). Prevalence, antimicrobial susceptibility and virulotyping of listeria species and *Listeria monocytogenes* isolated from open‐air fish markets. BMC Microbiology, 15, 1–7. 26209099 10.1186/s12866-015-0476-7 PMC4515007

[fsn34260-bib-0035] Karim, M. F. , Zhang, X. , & Li, R. (2019). Dynamics of shrimp farming in the southwestern coastal districts of Bangladesh using a shrimp yield dataset (SYD) and Landsat satellite archives. Sustainability, 11(17), 4635. 10.3390/su11174635

[fsn34260-bib-0036] Kaysner, C. A. , & DePaola, A., Jr. (2004). Laboratory methods – BAM: Vibrio. https://www.fda.gov/Food/FoodScienceResearch/LaboratoryMethods/ucm070830.htm

[fsn34260-bib-0038] Khan, M. , & Lively, J. A. (2023). Quality of farmed shrimp (*Penaeus monodon*, Fabricius, 1798 and *Macrobrachium rosenbergii*, deman, 1879) as affected by melanosis inhibiting compounds. Food Research, 7(4), 155–160.

[fsn34260-bib-0037] Khan, M. , Haque, A. , Rahman, M. M. , Paul, S. I. , Shaha, D. C. , Haque, F. , Sarkar, M. S. I. , & Shah, A. K. M. A. (2023). Antibiotic sensitivity of the bacteria isolated from pangas (*Pangasius hypophthalmus*) fish of the retail fish market of Gazipur, Bangladesh. Food Research, 7(6), 77–89.

[fsn34260-bib-0039] Khan, M. , Paul, S. I. , Rahman, M. M. , & Lively, J. A. (2022). Antimicrobial resistant bacteria in shrimp and shrimp farms of Bangladesh. Watermark, 14, 3172. 10.3390/w14193172

[fsn34260-bib-0040] Kishore, J. (2012). Isolation, identification & characterization of *Proteus penneri*–a missed rare pathogen. The Indian Journal of Medical Research, 135(3), 341–345. PMID: 22561620. 22561620 PMC3361870

[fsn34260-bib-0041] Lai, C. C. , Cheng, A. , Huang, Y. T. , Chung, K. P. , Lee, M. R. , Liao, C. H. , & Hsueh, P. R. (2011). *Escherichia fergusonii* bacteremia in a diabetic patient with pancreatic cancer. Journal of Clinical Microbiology, 49, 4001–4002. 10.1128/JCM.05355-11 21918030 PMC3209106

[fsn34260-bib-0042] Lakshmanan, R. , Jeya Shakila, R. , & Jeyasekaran, G. (2002). Survival of amine‐forming bacteria during the ice storage of fish and shrimp. Food Microbiology, 19(6), 617–625. 10.1006/fmic.2002.0481

[fsn34260-bib-0043] Leong, L. E. X. , Shaw, D. , Papanicolas, L. , Lagana, D. , Bastian, I. , & Rogers, G. B. (2017). Draft genome sequences of two *Enterobacter cloacae* subsp. *cloacae* strains isolated from Australian hematology patients with bacteremia. Genome Announcements, 5(3), pii:e00756‐17. 10.1128/genomeA.00756-17 PMC5604765 28818892

[fsn34260-bib-0044] Liu, L. , Lan, R. , Liu, L. , Wang, Y. , Zhang, Y. , Wang, Y. , & Xu, J. (2017). Antimicrobial resistance and cytotoxicity of Citrobacter spp. in Maanshan Anhui Province, China. Frontiers in Microbiology, 8, 1357. 10.3389/fmicb.2017.01357 28775715 PMC5518651

[fsn34260-bib-0045] Lohalaksanadech, D. , & Sajarit, C. (2015). Inspection of sanitary bacteria in the pacific white shrimp (*Litopeneaus vanamei*) pond culture, Trang province for developing a safety culture system. In 2015 international conference on science and technology (TICST) (pp. 98–100). IEEE.

[fsn34260-bib-0047] Mandal, S. C. , Hasan, M. , Rahman, M. S. , Manik, M. H. , Mahmud, Z. H. , & Islam, M. S. (2009). Coliform bacteria in Nile tilapia, *Oreochromis niloticus* of shrimp‐Gher, pond and fish market. World Journal of Fish and Marine Sciences, 1(3), 160–166. ISSN 1992–0083.

[fsn34260-bib-0046] Mandal, S. , Mandal, M. , Pal, N. , Halder, P. , & Basu, P. (2002). R‐factor in *Proteus vulgaris* from ulcerative disease of fish, *Channa punctatus* . Indian Journal of Experimental Biology, 40, 614–616. 12622213

[fsn34260-bib-0048] Marathe, N. P. , Gaikwad, S. S. , Vaishampayan, A. A. , Rasane, M. H. , Shouche, Y. S. , & Gade, W. N. (2016). Mossambicus tilapia (*Oreochromis mossambicus*) collected from water bodies impacted by urban waste carries extended‐spectrum beta‐lactamases and integron‐bearing gut bacteria. Journal of Biosciences, 41, 341–346. 27581926 10.1007/s12038-016-9620-2

[fsn34260-bib-0049] Matyar, F. (2017). Identification and antibiotic resistance of bacteria isolated from shrimps. In Proceedings of the International Journal of Arts & Sciences, Rome, Italy, 31 October–3 November 2017; pp. 289–294.

[fsn34260-bib-0081] McLauchlin, J. , Mitchell, R. T. , Smerdon, W. J. , & Jewell, K. (2004). *Listeria monocytogenes* and listeriosis: A review of hazard characterisation for use in microbiological risk assessment of foods. International Journal of Food Microbiology, 92(1), 15–33. 10.1016/s0168-1605(03)00326-x 15033265

[fsn34260-bib-0050] Messelhausser, U. , Colditz, J. , Tharigen, D. , Kleih, W. , Holler, C. , & Busch, U. (2010). Detection and differentiation of *Vibrio* spp. in seafood and fish samples with cultural and molecular methods. International Journal of Food Microbiology, 142, 360–364. 20688407 10.1016/j.ijfoodmicro.2010.07.020

[fsn34260-bib-0051] Mhango, M. , Mpuchane, S. F. , & Gashe, B. A. (2010). Incidence of indicator organisms, opportunistic and pathogenic bacteria in fish. African Journal of Food, Agriculture, Nutrition and Development, 10(10), 4202–4218. 10.4314/ajfand.v10i10.62898

[fsn34260-bib-0052] Mohammed, R. , Muhammed Ameen, S. , & Abdullah, S. (2023). Record of aerobic bacterial species from the cyprinid fish *Cyprinus Carpio* from fish farms in Sulaimani Province, Kurdistan region, Iraq. Applied Ecology and Environmental Research, 21(6), 5607–5624.

[fsn34260-bib-0053] Noor, R. , Hasan, M. F. , Munna, M. S. , & Rahman, M. M. (2015). Demonstration of virulent genes within Listeria and Klebsiella isolates contaminating the export quality frozen shrimps. International Aquatic Research, 7(2), 157–161. 10.1007/s40071-015-0097-7

[fsn34260-bib-0054] Norhana, M. W. , Poole, S. E. , Deeth, H. C. , & Dykes, G. A. (2010). Prevalence, persistence and control of Salmonella and Listeria in shrimp and shrimp products: A review. Food Control, 21(4), 343–361. 10.1016/j.foodcont.2009.06.020

[fsn34260-bib-0092] Noroozi, N. , Momtaz, H. , & Tajbakhsh, E. (2022). Molecular characterization and antimicrobial resistance of *Enterococcus faecalis* isolated from seafood samples. Veterinary Medicine and Science, 8(3), 1104–1112. 10.1002/vms3.761 35152566 PMC9122428

[fsn34260-bib-0090] Novais, C. , Campos, J. , Freitas, A. R. , Barros, M. , Silveira, E. , Coque, T. M. , Antunes, P. , & Peixe, L. (2018). Water supply and feed as sources of antimicrobial‐resistant *Enterococcus* spp. in aquacultures of rainbow trout (*Oncorhyncus mykiss*), Portugal. Science of the Total Environment, 625, 1102–1112. 10.1016/j.scitotenv.2017.12.265 29996407

[fsn34260-bib-0055] Novotny, L. , Dvorska, L. , Lorencova, A. , Beran, V. , & Pavlik, I. (2004). Fish: A potential source of bacterial pathogens for human beings. Veterinární Medicína, 49(9), 343–358. 10.17221/5715-VETMED

[fsn34260-bib-0056] Oh, J. Y. , Kang, M. S. , An, B. K. , Shin, E. G. , Kim, M. J. , Kwon, J. H. , & Kwon, Y. K. (2012). Isolation and epidemiological characterization of heat‐labile enterotoxin‐producing *Escherichia fergusonii* from healthy chickens. Veterinary Microbiology, 160, 170–175. 10.1016/j.vetmic.2012.05.020 22771038

[fsn34260-bib-0057] Parihar, V. S. , Barbuddhe, S. B. , Danielsson‐Tham, M. L. , & Tham, W. (2008). Isolation and characterization of Listeria species from tropical seafoods. Food Control, 19(6), 566–569. 10.1016/j.foodcont.2007.06.009

[fsn34260-bib-0058] Paul, S. I. (2018). Marine sponge and sponge associated bacteria inhibiting fish pathogen (pp. 1–94. MS thesis.). Bangabandhu Sheikh Mujibur Rahman Agricultural University.

[fsn34260-bib-0059] Preena, P. G. , Dharmaratnam, A. , Raj, N. S. , Raja, S. A. , Nair, R. R. , & Swaminathan, T. R. (2021). Antibiotic‐resistant Enterobacteriaceae from diseased freshwater goldfish. Archives of Microbiology, 203(1), 219–231. 32803348 10.1007/s00203-020-02021-8

[fsn34260-bib-0060] Pu, W. , Guo, G. , Yang, N. , Li, Q. , Yin, F. , Wang, P. , Zheng, J. , & Zeng, J. (2019). Three species of *Aeromonas* (*A. dhakensis*, *A. hydrophila* and *A. jandaei*) isolated from freshwater crocodiles (*Crocodylus siamensis*) with pneumonia and septicemia. Letters in Applied Microbiology, 68(3), 212–218. 30609084 10.1111/lam.13112

[fsn34260-bib-0085] Puah, S.‐M. , Puthucheary, S. D. , Liew, F.‐Y. , & Chua, K.‐H. (2013). *Aeromonas aquariorum* clinical isolates: Antimicrobial profiles, plasmids and genetic determinants. International Journal of Antimicrobial Agents, 41(3), 281–284. 10.1016/j.ijantimicag.2012.11.012 23312608

[fsn34260-bib-0088] Pérez, L. , Abarca, M. , Latif‐Eugenín, F. , Beaz‐Hidalgo, R. , Figueras, M. , & Domingo, M. (2015). *Aeromonas dhakensis* pneumonia and sepsis in a neonate Risso's dolphin *Grampus griseus* from the Mediterranean Sea. Diseases of Aquatic Organisms, 116(1), 69–74. 10.3354/dao02899 26378409

[fsn34260-bib-0061] Qasim, D. A. (2023). Isolation and identification of some types of histamine‐producing bacteria from shrimp in local markets. Revis Bionatura, 28(1), 47. 10.21931/RB/CSS/2023.08.01.47

[fsn34260-bib-0062] Rahimi, E. , Raissy, M. , Razzaghimanesh, M. , Dastgerdi, A. , & Shahraki, M. (2014). Occurrence of *Aeromonas hydrophila* in fish, shrimp, lobster and crab in Iran. Kafkas Üniversitesi Veteriner Fakültesi Dergisi, 20(5), 691–696. 10.9775/kvfd.2014.10892

[fsn34260-bib-0065] Rahman, M. M. , Hosen, M. J. , Rahman, M. H. , & Azad, M. (2012). Bacteriological quality of fresh shrimps of natural and artificial habitats in Khulna District. Bangladesh. International Journal of Bioscience, 10, 61–65. ISSN: 2220‐6655.

[fsn34260-bib-0063] Rahman, M. , Rahman, M. M. , Deb, S. C. , Alam, M. S. , Alam, M. J. , & Islam, M. T. (2017). Molecular identification of multiple antibiotic resistant fish pathogenic *Enterococcus faecalis* and their control by medicinal herbs. Scientific Reports, 7(1), 1–11. 10.1038/s41598-017-03673-1 28623336 PMC5473830

[fsn34260-bib-0064] Rahman, M. , Somsiri, T. , Tanaka, R. , Sawabe, T. , & Tajima, K. (2005). PCR‐RFLP analysis for identification of Aeromonas isolates collected from diseased fish and aquatic animals. Fish Pathology, 40(4), 151–159. 10.3147/jsfp.40.151

[fsn34260-bib-0066] Ray, B. , & Bhunia, A. (2007). Fundamental food microbiology. CRC Press.

[fsn34260-bib-0067] Rayamajhi, N. , Cha, S. B. , Shin, S. W. , Jung, B. Y. , Lim, S. K. , & Yoo, H. S. (2011). Plasmid typing and resistance profiling of *Escherichia fergusonii* and other *Enterobacteriaceae* isolates from south Korean farm animals. Applied and Environmental Microbiology, 77, 3163–3166. 10.1128/AEM.02188-10 21398479 PMC3126392

[fsn34260-bib-0068] Rimoldi, G. M. , & Moeller, R. B., Jr. (2013). *Escherichia fergusonii* associated with pneumonia in a beef cow. Journal of Veterinary Medicine, 2013, 829532. 10.1155/2013/829532 26464912 PMC4590865

[fsn34260-bib-0069] Roy, D. , Biswas, B. , Islam, H. R. , Ahmed, M. S. , Rasheduzzaman, M. , & Sarower, M. G. (2013). Rapid identification of Enterovirulent *Escherichia coli* strains using polymerase chain reaction from shrimp farms. Pakistan Journal of Biological Sciences: PJBS, 16, 1260–1269. 10.3923/pjbs.2013.1260.1269 24511733

[fsn34260-bib-0070] Saha, S. , Halder, M. , Mookerjee, S. , & Palit, A. (2020). Preponderance of multidrug‐resistant, toxigenic, and Thermotolerant Enteropathogenic bacteria in raw and cooked seafood of indo‐Gangetic Basin and associated health risks. Journal of Aquatic Food Product Technology, 29(9), 838–849. 10.1080/10498850.2020.1813858

[fsn34260-bib-0071] Sheng, L. , & Wang, L. (2021). The microbial safety of fish and fish products: Recent advances in understanding its significance, contamination sources, and control strategies. Comprehensive Reviews in Food Science and Food Safety, 20(1), 738–786. 10.1111/1541-4337.12671 33325100

[fsn34260-bib-0089] Shikongo‐Nambabi, M. N. N. N. (2011). Occurance and control of *Vibrio* species as contaminants of processed marine fish. [Doctoral dissertation]. University of Pretoria.

[fsn34260-bib-0086] Soto‐Rodriguez, S. A. , Cabanillas‐Ramos, J. , Alcaraz, U. , Gomez‐Gil, B. , & Romalde, J. L. (2013). Identification and virulence of *Aeromonas dhakensis*, *Pseudomonas mosselii* and *Microbacterium paraoxydans* isolated from *Nile tilapia*, *Oreochromis niloticus*, cultivated in Mexico. Journal of Applied Microbiology, 115(3), 654–662. 10.1111/jam.12280 23758410

[fsn34260-bib-0072] Surendraraj, A. , Thampuran, N. , & Joseph, T. C. (2010). Molecular screening, isolation, and characterization of enterohemorrhagic *Escherichia coli* O157:H7 from retail shrimp. Journal of Food Protection, 73, 97–103. 10.4315/0362-028x-73.1.97 20051211

[fsn34260-bib-0073] Tsironi, T. N. , & Taoukis, P. S. (2018). Current practice and innovations in fish packaging. Journal of Aquatic Food Product Technology, 27(10), 1024–1047. 10.1080/10498850.2018.1532479

[fsn34260-bib-0074] U.S. Food and Drug Administration . (2018). Refusal actions by FDA as recorded in OASIS for 16‐fishery/seafood product. Retrieved August 23, 2018, from https://www.accessdata.fda.gov/scripts/importrefusals/

[fsn34260-bib-0075] U.S. Food and Drug Administration . (2019). Refusal actions by FDA as recorded in OASIS for 16‐fishery/seafood prod‐uct. Retrieved March 31, 2020, from https://www.accessdata.fda.gov/scripts/importrefusals/

[fsn34260-bib-0076] Velu, S. , Cheong Yew, C. , Zaman, M. Z. , & Abu Bakar, F. (2019). Inhibition of melanosis, microbial and quality changes of white shrimp (*Penaues vannameii*) via effect of key lime juice and vacuum packaging at 2±1°C. Journal of Aquatic Food Product Technology, 28(4), 427–437. 10.1080/10498850.20

[fsn34260-bib-0093] World Health Organization . (2020). Risk assesment tools for Vibrio parahaemolyticus and Vibrio vulnificus associated with seafood .

[fsn34260-bib-0087] Wu, C.‐J. , Chen, P.‐L. , Hsueh, P.‐R. , Chang, M.‐C. , Tsai, P.‐J. , Shih, H.‐I. , Wang, H.‐C. , Chou, P.‐H. , & Ko, W.‐C. (2015). Clinical implications of species identification in monomicrobial *Aeromonas* bacteremia. PLoS One, 10(2), e0117821. 10.1371/journal.pone.0117821 25679227 PMC4334500

[fsn34260-bib-0077] Zheng, W. , Yang, X. , Lu, L. , He, S. , Jian, A. , & Cao, H. (2015). *Aeromonas schubertii*: A potential pathogen for freshwater cultured whiteleg shrimp, *Penaeus vannamei* . Israeli Journal of Aquaculture – Bamidgeh, 67, 20732.

